# Bilateral simple ectopic kidney in a cat 

**Published:** 2017-06-15

**Authors:** Masoud Rajabioun, Hamideh Salari Sedigh, Ali Mirshahi

**Affiliations:** *Department of Clinical Sciences, Faculty of Veterinary Medicine, Ferdowsi University of Mashhad, Mashhad, Iran*

**Keywords:** Cat, Domestic Short Hair, Ectopic kidney, Radiology, Ultrasonography

## Abstract

Bilateral simple ectopic kidney was diagnosed in an apparently healthy 3-year-old, female domestic short hair cat, incidentally based on radiology and ultrasonography examination. The cat was presented for routine examination without any complaint. In clinical evaluation, no significant abnormal clinical sign was seen except for the absence of both kidneys in their proper location in abdominal palpation, which they were palpated more caudally. Radiography revealed silhouettes of soft tissue opacity in the caudal part of the abdominal cavity superimposed on urinary bladder. Ultrasonography confirmed the presence of both kidneys more caudally. The left kidney was dorsal to the urinary bladder and the right kidney located cranially than the left one. Each kidney showed normal shape and size and imaged in their proper side. Hematological, biochemical and urinalysis examinations showed normal values. Ectopic kidney can be diagnosed in feline patients as an incidental finding but it is important to evaluate the kidney for concurrent problems.

## Introduction

Ectopia is the congenital malposition of one or both kidneys.^1^ Ectopic kidney is classified into simple and cross according to the human literature. In simple ectopic kidney, ureter and vesico-ureteral junction remain in the ipsilateral retroperitoneal space but in cross ectopic kidney, kidney is located on the opposite site and the related ureter crosses the midline.^2^^,^^3^ Simple ectopic kidney is usually asymptomatic condition and rare congenital malformation.^4^^-^^7^ In literature review this abnormality have been reported in dogs,^4^^,^^6^^,^^8^ cats,^5^^-^^7^^,^^9^^-^^11^ swines^12^ and calves.^13^ Radiography, ultrasonography and intravenous pyelo-graphy (IVP) are usually used for diagnosis.^5^^,^^9^^,^^10^

Etiology of the renal ectopia is unclear.^3^^,^^9^ Two theories have been described about the underlying causes interfering with normal ascent of kidneys including failed growth or development of the ureteric bud or metanephric mesenchyme due to damage during primordial renal tissue formation and keeping the kidney in abnormal position because of mechanical resistance from adjacent structures.^9^

Hydronephrosis, infection and calculi can be occurred because of poor urine outflow.^2^^,^^3^ Hydronephrosis is the most common finding in humans with renal ectopia.^2^

In veterinary medicine, most of the ectopic kidneys were diagnosed incidentally because of no specific associated clinical signs.^3^^,^^5^^,^^6^^,^^8^^,^^10^^,^^14^ Fusion of both kidneys is another congenital abnormality can be associated with renal ectopia.^9^^,^^15^

## Case Description

**Fig. 1:**
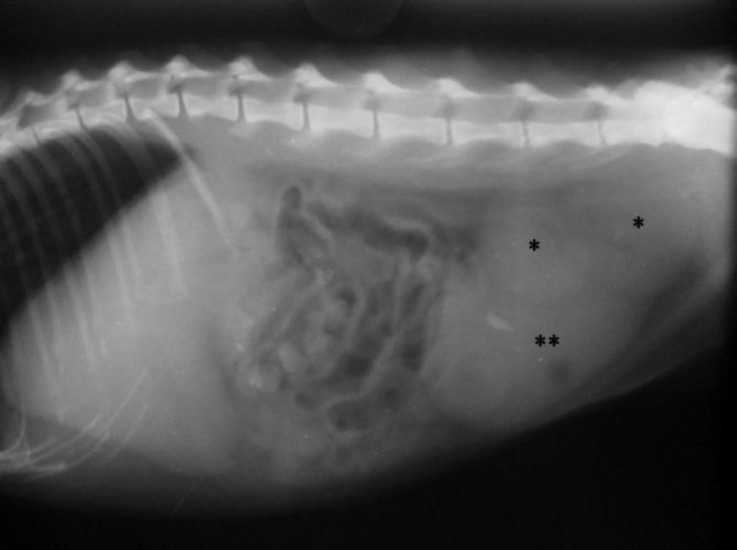
Lateral abdominal radiograph. Note the absence of both renal silhouettes in their proper location and presence of the soft tissue opacities in the caudal part of the abdomen (*) superimposed on the urinary bladder silhouette (**).

**Fig. 2:**
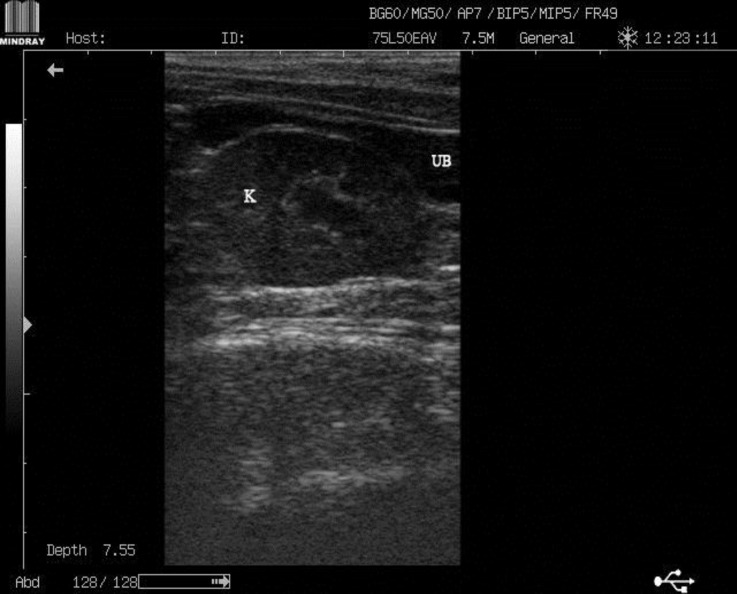
Longitudinal ultrasonographical image of the caudal part of the abdomen. The left kidney was imaged in dorsal aspect of the urinary bladder nearly in the midline. K: Kidney, UB: Urinary bladder. Cranial part of the abdomen is on the left

**Fig. 3.**
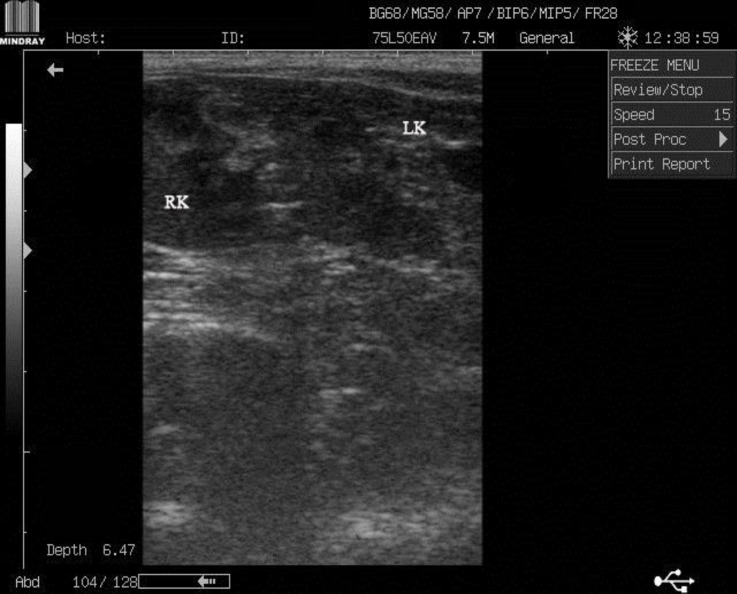
Oblique longitudinal ultrasonographical image of the caudal part of the abdomen. Both kidneys were imaged simultaneously. The right and the left kidneys are on the left and right sides of the image, respectively. Part of urinary bladder was imaged on the left side. LK: Left kidney, RK: Right kidney. Cranial part of the right side of abdomen is on the left

A 3-year-old, female, domestic short hair (DSH) cat was presented to the veterinary teaching hospital, Ferdowsi University of Mashhad for routine examination. In clinical examinations, cat showed normal body condition and no clinical sign was observed. On abdominal palpation, both kidneys were not detectable in their proper location and two non-painful symmetrical structures were palpated in the caudal part of the abdomen. For further evaluation, the cat was referred to diagnostic imaging section for radiological and ultrasonographical examinations. On lateral radiograph, renal silhouette was not seen in the true location and the soft tissue opacities were detected in the caudal part of the abdomen at the level of L5-L7 superimposed on the urinary bladder silhouette (Fig. 1). Ultrasonographical examination confirmed the radio-logical findings. Left kidney was imaged in the caudal part of the abdomen, dorsal to urinary bladder on the left side near the midline (Fig. 2). Right kidney was detected slightly cranial to the left kidney on the right side of the abdomen; both kidneys were imaged by oblique longitudinal plane simultaneously (Fig. 3).

Both kidneys’ hili were imaged in medial aspect and both kidneys showed normal shape, size and architecture, ultrasonographically. Mild increased renal cortical echogenicity was seen in both kidneys. No significant abnormalities were detected following hematological, biochemical and urinalysis evaluations. 

Further investigation was required for performing IVP procedure under general anesthesia but the cat’s owner refused it because the cat only was represented for a normal clinical examination.

After six months, the cat was in really good condition without any abnormal clinical or ultrasonographical sign. Unfortunately, the next follow up could not be done because the cat ran away from home.

## Discussion

In this report, based on radiographical and ultrasono-graphical findings, bilateral simple ectopic kidney was diagnosed incidentally in a 3-year-old DSH cat without any clinical sign or other congenital malformation.

Normally, kidneys are positioned within the retro-peritoneal space. The left kidney lies ventral to the first three lumbar vertebrae and the right kidney a half vertebral length more cranial.^16^ Ectopic kidney has been reported in dogs, cats, swine and calves.^4^^-^^13^ In cat, all of the reports about the simple ectopic kidney were unilateral and this is the first report of bilateral simple ectopic kidney in DSH cat. The right kidney was more affected than the left kidney and no sex predilection was reported.^6^^,^^10^ Although, ectopic kidney diagnosis was done usually based on radiography, ultrasonography and intravenous uro-graphy,^5^^,^^9^^,^^10^ unfortunately we could not persuade the cat’s owner to perform intravenous urography because the cat was clinically normal. Ultrasonographical examination revealed that each kidney was located in its proper side because the kidneys hili were imaged medially.

Poor outflow with subsequent predisposition to hydronephrosis, infection and calculi can be occurred because of malposition of the kidneys. Ectopic kidney is more susceptible to infection and obstruction.^2^^,^^3^ Because of malposition of one or both kidneys, hydronephrosis may be developed due to impaired urinary drainage.^14^^,^^17^ The most common and significant finding in humans with renal ectopia is hydronephrosis and associated vesico ureteral reflux is estimated in 25 to 70% of cases.^2^ In this case both kidneys were imaged completely by ultrasonography and no sign of hydronephrosis or calculi was seen. Urinary bladder wall and its contents showed normal ultrasonographical appearance. In follow up ultrasono-graphical examination after six months, abnormal findings were not detected.
